# Population-based study of genetic variation in individuals with autism spectrum disorders from Croatia

**DOI:** 10.1186/1471-2350-11-134

**Published:** 2010-09-21

**Authors:** Li-San Wang, Dubravka Hranilovic, Kai Wang, Ingrid E Lindquist, Lindsay Yurcaba, Zorana-Bujas Petkovic, Nicole Gidaya, Branimir Jernej, Hakon Hakonarson, Maja Bucan

**Affiliations:** 1 Departments of Pathology and Laboratory Medicine, Biomedical Graduate Studies, University of Pennsylvania, Philadelphia, 19104, USA; 2 Penn Center for Bioinformatics, Biomedical Graduate Studies, University of Pennsylvania, Philadelphia, 19104, USA; 3 Faculty of Science, University of Zagreb; Institute "Rudjer Boskovic, Zagreb, Croatia; 4 Center for Applied Genomics, The Children's Hospital of Philadelphia, 19104, USA; 5 Genetics, Biomedical Graduate Studies, University of Pennsylvania, Philadelphia, 19104, USA; 6 Neuroscience Graduate Group - Biomedical Graduate Studies, University of Pennsylvania, Philadelphia, 19104, USA; 7 Psychiatric Hospital for Children and Youth, Zagreb, Croatia, Institute "Rudjer Boskovic, Zagreb, Croatia; 8 Pediatrics, Biomedical Graduate Studies, University of Pennsylvania, Philadelphia, 19104, USA; 9 Laboratory for Neurochemistry and Molecular Neurobiology, Institute "Rudjer Boskovic, Zagreb, Croatia

## Abstract

**Background:**

Genome-wide studies on autism spectrum disorders (ASDs) have mostly focused on large-scale population samples, but examination of rare variations in isolated populations may provide additional insights into the disease pathogenesis.

**Methods:**

As a first step in the genetic analysis of ASD in Croatia, we characterized genetic variation in a sample of 103 subjects with ASD and 203 control individuals, who were genotyped using the Illumina HumanHap550 BeadChip. We analyzed the genetic diversity of the Croatian population and its relationship to other populations, the degree of relatedness via Runs of Homozygosity (ROHs), and the distribution of large (>500 Kb) copy number variations.

**Results:**

Combining the Croatian cohort with several previously published populations in the FastME analysis (an alternative to Neighbor Joining) revealed that Croatian subjects cluster, as expected, with Southern Europeans; in addition, individuals from the same geographic region within Europe cluster together. Whereas Croatian subjects could be separated from a sample of healthy control subjects of European origin from North America, Croatian ASD cases and controls are well mixed. A comparison of runs of homozygosity indicated that the number and the median length of regions of homozygosity are higher for ASD subjects than for controls (p = 6 × 10^-3^). Furthermore, analysis of copy number variants found a higher frequency of large chromosomal rearrangements (>2 Mb) in ASD cases (5/103) than in ethnically matched control subjects (1/197, p = 0.019).

**Conclusions:**

Our findings illustrate the remarkable utility of high-density genotype data for subjects from a limited geographic area in dissecting genetic heterogeneity with respect to population and disease related variation.

## Background

Autism Spectrum Disorders (ASD, MIM209850) are a severe neuropsychiatric disorders, primarily characterized by abnormalities in social behavior, communication and language, with patterns of restricted and repetitive interests [1]. Commonly associated symptoms include aggressive and self-injurious behaviors, anxiety, heightened sensitivity to stimuli, and seizures [2,3]. Severity of symptoms in ASD can vary widely among cases, from individuals with mental retardation and no language production to relatively high-functioning individuals with normal to superior intelligence but significant difficulties with social interaction [4-6].

Multiple lines of evidence converge to suggest that ASDs represent the most heritable neurodevelopmental and psychiatric conditions. Evidence from twin and family studies suggests that the rate of autism in siblings of affected individuals is 2-6% [7], with 92% concordance of ASD in monozygotic twins and over 10% concordance in dizygotic twins [8]. Previous linkage and candidate gene studies have identified several chromosomal regions with autism susceptibility loci [3,9-13]. A recent study identified a common genetic risk factor underlying ASD employing a genome-wide association strategy in 780 families (3,101 subjects) with affected children, and a second cohort of 1,204 affected subjects and 6,491 control subjects; these findings were replicated in an independent study cohort of 483 ASD families [14]. Furthermore, substantial progress towards the identification of genetic risk variants has come from recent characterization of structural variation (*i.e.*, copy number variation or CNV). Studies of CNVs in a cohort of 859 ASD cases and 1,400 healthy control children revealed structural variants in previously reported ASD candidate genes (*NRXN1 *and *CNTNAP4*) and in multiple novel susceptibility genes encoding cell-adhesion molecules, including *NLGN1 *and *ASTN2*. Furthermore, CNVs observed in cases but not controls were found within and surroundings genes involved in the ubiquitin pathway (*UBE3A*, *PARK2*, *RFWD2 *and *FBXO40*) [15]. In a related study, we performed high-density genotyping and obtained data on 3,832 individuals from 912 multiplex ASD families in the Autism Genetic Research Exchange (AGRE) and 1,489 unrelated control subjects. Through prioritization of exonic deletions (eDels), we recovered genes in which structural variants were present in multiple unrelated probands but not unrelated controls. The 42 genes identified by this method include *NRXN1*, *UBE3A, MADCAM1 *and *BZRAP1 *[16]. Studies of large families with shared ancestry have reported several other autism loci including large, inherited, homozygous deletions in neuronal cell-adhesion genes, for example *PCDH10 *(protocadherin 10) [17]. These findings highlight the utility of "homozygosity mapping" and a need to search for additional large families across diverse populations.

Based on these breakthroughs and lessons learned from studies of other complex diseases (i.e. diabetes and breast cancer), it is becoming apparent that there is a need to assess large sample collections that number in the tens of thousands. Population structure poses a challenge for large-scale disease-association studies; mismatched ancestry between cases and controls in a genome-wide association studies is a potential source of spurious associations [17]. These needs and challenges bring together usually separated fields of genetics, epidemiology and studies of human diversity. Also, it is important to relate genetic findings found in patients from developed countries to the studies of diverse populations in less developed parts of the world. Currently DNA has been assembled from only a few thousand individuals with autism worldwide. Significant advances in genetic analyses of ASD in diverse populations will require the collection of samples across many populations using rapid and affordable screening and diagnostic tools.

Isolated populations have contributed to the discovery of genetic factors for many Mendelian disorders [18]. It has been argued that isolated populations may facilitate the identification of susceptibility loci for complex diseases because of a reduced genetic diversity, i.e. reduced genetic heterogeneity [19,20]. Although several Croatian island populations have been described as genetic isolates, the Croatian population as a whole does not represent a population isolate as conventionally defined [21,22]. However, Croatia is a small country with a low migration rate, in which large extended families often live in the same household or same neighbourhood and, therefore, is perfectly suited for family-based genetic studies. We expect that a subset of affected individuals may segregate a limited number of causative ASD susceptibility alleles (causal variants); in some cases these loci or disease-associated haplotypes identified in genetic isolates may differ from those in more genetically diverse populations, such as Europeans in North America. We also expect that a subset of rare variants associated with ASD susceptibility (SNPs and CNVs), which were identified in a large set of Europeans from North America, may be more prevalent in specific ethnic groups like the Croats. Therefore, to improve our understanding of global patterns of human genetic variation and set the stage for large-scale disease studies, efforts are underway to evaluate genetic variation using high-density genotype data for human populations worldwide [23-25].

With the intention to use the Croatian population in future genome-wide association studies, we performed a genome-wide high-density genotype pilot study of 103 ASD cases and 203 control samples to characterize the genetic structure of the population. Our analysis focuses on (1) the genetic diversity of the Croatian population, and its relationship to other populations, (2) the degree of relatedness of the population through the analysis of Runs of Homozygosity (ROHs), and (3) the distribution of large (>500 Kb) copy number variations, a major mechanism for structural variation in human genomes, and its possible association with ASD.

## Results and Discussion

To understand the complexities of genomic architecture of ASDs, a highly heterogeneous group of disorders, and to assess the distribution and specificity of common and rare variants in different populations, we initiated genomic analysis of ASD and control subjects from Croatia. This cohort, consisting of 103 subjects (children and adults) with ASD and 203 adult control subjects, with male-to-female ratios of 3.7:1 (in ASD) and 2.6:1 (in controls), was originally recruited to study the role of hyperserotonemia in autism [26-28]. Patients with autism were evaluated by a child psychiatrist and diagnoses were based on DSM-IV-TR [29] criteria. Severity of behavioral symptoms was measured using the Childhood Autism Rating Scale (CARS) [30]. The degree of mental retardation (MR) was assessed according to the standardized intelligence or developmental tests, corresponding to the apparent developmental level of each individual. ASD in selected families occurs either in the absence of positive family history (sporadic) or in families with a high prevalence of other clinical psychiatric disorders distinct from ASD (such as anxiety, depression and phobias). During the clinical assessment and interviews, psychiatrists and geneticists did not notice any recognizable dysmorphic features in selected study subjects. DNA samples isolated from whole blood were genotyped with the Illumina HumanHap550 SNP array and 306 samples gave high quality data that were subjected to population structure and CNV analysis. See Table 1 for a summary of the genome-wide datasets used in this study.

**Table 1:** Summary of datasets with whole-genome genotyping

Dataset		#Subjects	#Case	Platform	#SNPs
**Croatia**	*(Before QC)*	306	103	Illumina 550 K	561,466
	*(After QC)*	305	103		489,200
**NINDS**	*(Before QC)*	542	0	Illumina 550 K	561,466
	*(After QC)*	534	0		489,096
**HGDP**	*(Before QC)*	1,043	0	Illumina 650Y	660,918
	*(After QC)*	1,043	0		584,835

		*Merged datasets*			

**Croatia+NINDS**	*(After QC)*	839	103	-	475,964
**Croatia+HGDP**	*(After QC)*	1,348	103	-	478,287

**Figure 1:**
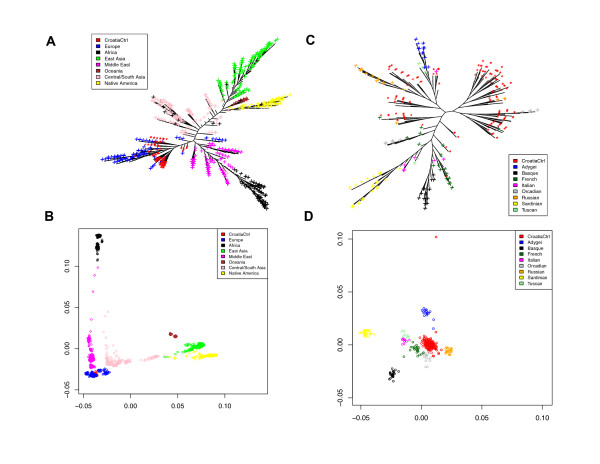
**Population structure of Croatia Cohort**. (A) FastME tree using IBS distance and (B) IBS distance multidimensional scaling (MDS) for Croatia control + HGDP, colored by HGDP region IDs. (C) FastME tree using IBS distance and (D) IBS distance multidimensional scaling (MDS) for Croatia Control + HGDP European Only, colored by HGDP subpopulation IDs.

**Figure 2:**
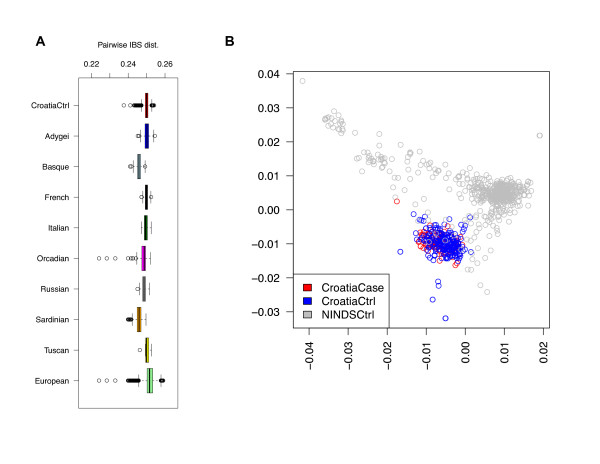
**Pairwise IBS analysis**. (A) Box-and-whisker plot of pairwise IBS distances for Croatia Control, subpopulations of HGDP European Cohort, and the whole HGDP European Cohort. (B) Scatter plot of the first two coordinates of IBS distance multidimensional scaling (MDS) for Croatia control, case, and NINDS control cohorts.

To understand the genetic homogeneity of the Croatian cohort and its position in the worldwide human population, we used phylogenetic tree analysis, principal component analysis (PCA) (not shown), and multi-dimensional scaling (MDS), three tools commonly used in the study of human population genetic diversity [23,24]. We used data from the Human Genome Diversity Project (HGDP), which consists of genome-wide SNP data of 1007 individuals from 51 populations, divided into 7 geographic regions. Of these individuals, 153 were from 8 populations from the European region. We plotted the results of MDS and tree analyses using control subjects from Croatia and the HGDP samples (Figure 1). All three methods show the same overall pattern emerging from previous HGDP studies: the relative positions of the major regions largely correlate with their relative geographical locations, and reflect the human migration history well. Humans migrated from Africa to the Middle East (Figure 1A-B); the first branching took place, and one group migrated to Europe, while the other migrated to South Asia. A second major branching then occurred, with one migration to America (through Alaska), the other countinuing to East Asia and eventually Oceania. As expected, the Croatia control samples are entirely contained within the European samples. A focused analysis of Europeans in the HGDP panel (Figure 1C-D) with the Croatia control samples situated in the middle of phylogeny or MDS plots shows the relationship of this subpopulation to other European populations. The Sardinians and the Basques seem to be the farthest, with Italian samples placed in between them and the Croatian controls; the other subpopulation that is separate from the Croatia controls is the Adygei. The Croatians are most similar to the Russians and the Orcadians. Both the PCA (not shown) and MDS plots show the relative diversity of the Croatian controls (indicated by the area spanned by the samples on the MDS and PCA plots) to be similar to the other populations. The boxplot of pairwise Identity by State (IBS) distances for European populations (Figure 2A) showed the Croatian control cohort has divergence similar to other European subpopulations (except Basques and Sardinians who are genetically more homogeneous), and smaller than the European population as a whole.

**Figure 3:**
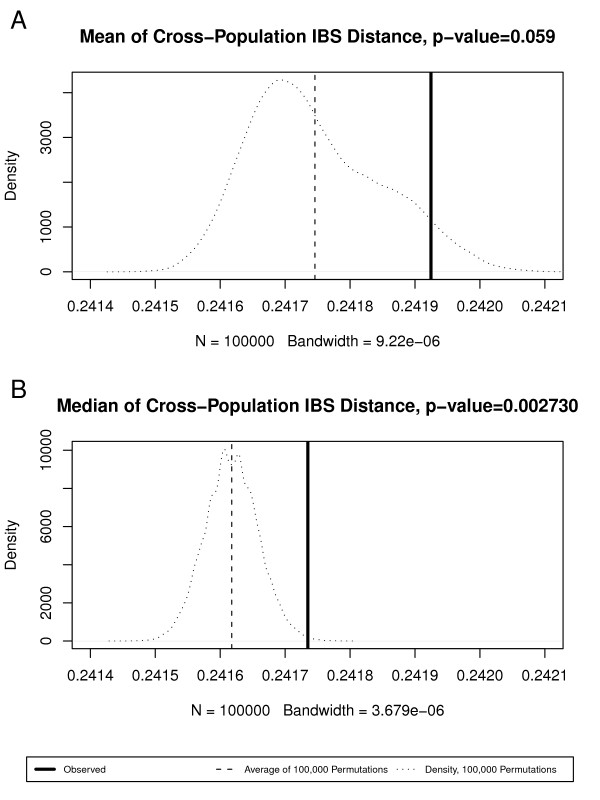
**Analysis of median and mean cross-population pairwise IBS distances between Croatia Control and NINDS**. (A) Observed median IBS distance and distribution of median IBS distances by random shuffling of population memberships; (B) observed mean IBS distance and distribution of median IBS distances by random shuffling of population memberships.

We performed MDS on the Croatian controls and cases, as well as NINDS (National Institute of Neurological Disorders and Stroke) control cohorts to check the relationships between the three cohorts (Figure 2B), and found that the Croatian control and cases are well mixed but separate from the NINDS controls. Randomization tests by shuffling between Croatian control and NINDS controls showed the median pairwise IBS (identity-by-state) distance across the two subsets (0.28) is significantly higher than expected by chance (p = 0.00273, see Figure 3); this agrees with the MDS visualization that the two cohorts are genetically separate. Analysis of fixation index (F_ST_) led to the same conclusion (see Table 2 for top regions with large F_ST_). In summary, the Croatian and the NINDS cohorts have significant genetic difference, and care must be taken to correct for population stratification in combining the two datasets for association studies.

**Table 2:** Top windows for sliding window F_ST _analysis

Cytoband	Coordinate	#SNP	Mean Fst	Median Fst	Window/genomic ratio of Mean Fst	Window/genomic ratio of Median Fst
1q23.2-1q23.3	chr1:157.5-162.5 Mb	1031	0.00170	0.00078	1.47	1.51
1q31.3	chr1:192.5-197.5 Mb	663	0.00186	0.00064	1.61	1.23
2q21.2-2q22.1	chr2:132.5-137.5 Mb	854	0.00242	0.00105	2.10	2.02
2q21.3-2q22.1	chr2:135-140 Mb	653	0.00233	0.00089	2.02	1.71
3q26.1-3q26.2	chr3:165-170 Mb	592	0.00185	0.00101	1.60	1.95
4q31.21-4q31.22	chr4:142.5-147.5 Mb	571	0.00236	0.00124	2.05	2.38
4q31.22-4q31.23	chr4:145-150 Mb	644	0.00187	0.00078	1.62	1.51
5q21.3-5q22.1	chr5:105-110 Mb	866	0.00167	0.00078	1.45	1.51
5q22.1-5q22.3	chr5:110-115 Mb	930	0.00155	0.00083	1.34	1.59
5q31.1-5q31.3	chr5:135-140 Mb	599	0.00220	0.00084	1.91	1.62
6p22.2-6p21.33	chr6:25-30 Mb	951	0.00182	0.00081	1.58	1.56
6p22.1-6p21.32	chr6:27.5-32.5 Mb	1339	0.00280	0.00140	2.42	2.69
6p21.33-6p21.31	chr6:30-35 Mb	1524	0.00285	0.00133	2.47	2.57
6p21.32-6p21.2	chr6:32.5-37.5 Mb	1096	0.00204	0.00084	1.77	1.62
6p12.1-6p11.1	chr6:55-60 Mb	440	0.00162	0.00079	1.40	1.51
6q11.1-6q12	chr6:62.5-67.5 Mb	618	0.00168	0.00094	1.45	1.81
6q16.3-6q21	chr6:100-105 Mb	748	0.00157	0.00085	1.36	1.63
7q31.32-7q32.1	chr7:122.5-127.5 Mb	776	0.00143	0.00084	1.24	1.61
8q21.13-8q21.3	chr8:82.5-87.5 Mb	590	0.00143	0.00083	1.24	1.60
8q21.2-8q21.3	chr8:85-90 Mb	626	0.00201	0.00098	1.74	1.88
9q12-9q21.11	chr9:67.5-72.5 Mb	414	0.00170	0.00088	1.48	1.69
9q33.2-9q33.3	chr9:122.5-127.5 Mb	681	0.00156	0.00080	1.35	1.54
9q33.2-9q34.11	chr9:125-130 Mb	804	0.00157	0.00079	1.36	1.52
11p11.2-11p11.12	chr11:45-50 Mb	443	0.00177	0.00062	1.54	1.19
11q13.1-11q13.3	chr11:65-70 Mb	597	0.00170	0.00083	1.48	1.61
12p11.1-12q12	chr12:35-40 Mb	540	0.00148	0.00100	1.28	1.93
13q31.1-13q31.3	chr13:85-90 Mb	688	0.00156	0.00085	1.35	1.63
20q11.21-20q11.23	chr20:30-35 Mb	461	0.00163	0.00093	1.41	1.78
22q13.1-22q13.2	chr22:37.5-42.5 Mb	757	0.00159	0.00080	1.38	1.54

Mapping of large regions of homozygosity in pedigrees with shared ancestry has been employed in the discovery of autosomal recessive diseases associated with neurodevelopmental anomalies [31-33], and more recently, in the identification of novel genes associated with ASD, schizophrenia and Alzheimer's Disorder [17,34,35]. Although we recruited unrelated ASD cases and healthy controls, we analyzed genome-wide rates of homozygosity to estimate consanguinity and compared these rates to a heterogeneous control sample of Europeans from North America. It has been suggested that mapping of extended tracks of homozygosity may facilitate identification of low-frequency variants associated with complex diseases [36,37]. We detected regions ("runs") of homozygosity (ROH) using the PLINK v1.05 software [38] and compared the extent of homozygosity in Croatia ASD cases, control subjects and NINDS controls. We computed the number of ROH segments, total ROH length, and average ROH length for the three subsets (Table 3). We detected subtle differences between the three groups for the median number of ROHs (Kruskal-Wallis test; p = 0.06), as well as average ROH length between Croatia controls and NINDS controls (Wilcoxon test; p = 0.053), with NINDS cohort showing shorter total ROH length than Croatia controls (Kruskal-Wallis test; p = 0.0056, higher due to the very large number of NINDS samples). However, the most significant pairwise difference is the total ROH length between Croatian cases Croatian controls (Wilcoxon test; p = 0.0065).

**Table 3:** Summary of the ROH comparison across Croatia Control, Croatia Case, and NINDS sample groups.

		#ROHs	Total ROH length(KB)	Average ROH length (KB)
*Median*				

	Croatia Ctrl	15	23486.3	1512.2
	Croatia Case	16	26144	1519.3
	NINDS	16	23909.3	1476.3

*Wilcoxon Test P-value*				

	Croatla Ctrl vs NINDS	0.0346	0.3928	0.0529
	Croatla Case vs Ctrl	0.0413	**0.0065**	0.17
	Croatla Case vs NINDS	0.6115	0.0248	**0.0037**

*Kruskal-Wallis Test P-value*				

	All three groups	0.0604	0.0279	**0.0056**

**Figure 4:**
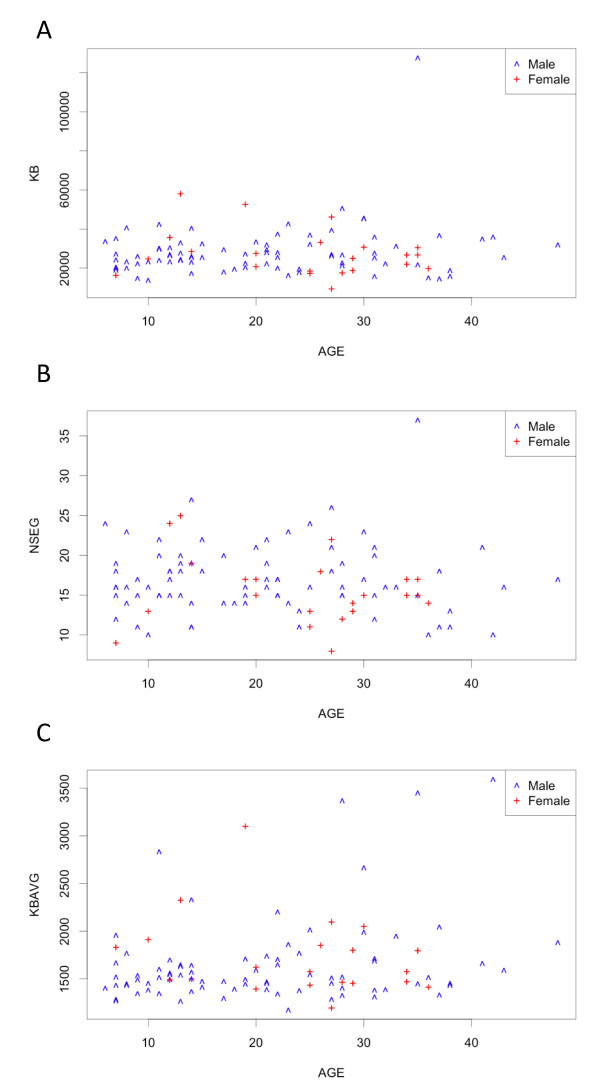
**Scatter plots for ROH number and length versus Age of Subject**. (A) Total ROH length (in Kb) versus Age; (B) Number of ROHs versus Age; (C) Average ROH size (in Kb) versus Age.

Motivated by the study demonstrating the positive correlation between runs of homozygosity and age in a North American cohort of European descent due to urbanization [35], we examined age correlation with ROH frequency and size in the 103 ASD subjects (Figure 4 and Table 4). We found only borderline significance between age and average ROH size, though the lack of significance may be due to the fact that the Croatia ASD cohort is 40 years younger (mean age is 21.5 y as opposed to 61.7 y in Nalls et al. [35]).

**Table 4:** Correlation between ROH and age

		No. ROH	Total ROH Size (Kb)	Average ROH Size (Kb)
**(Intercept)**	*Coefficient*	17.8	35677.3	2009.64
	*Coefficient*	-0.09	-328.5	-11.25
**AGE**	*P-value*	*0.409*	*0.322*	*0.303*
	*Coefficient*	-0.71	-12177.4	-605.62
**SEX(male)**	*P-value*	*0.815*	*0.185*	***0.0463***
	*Coefficient*	0.087	564.4	23.26
**AGE:SEX(male)**	*P-value*	*0.467*	*0.119*	***0.0517***

**Figure 5:**
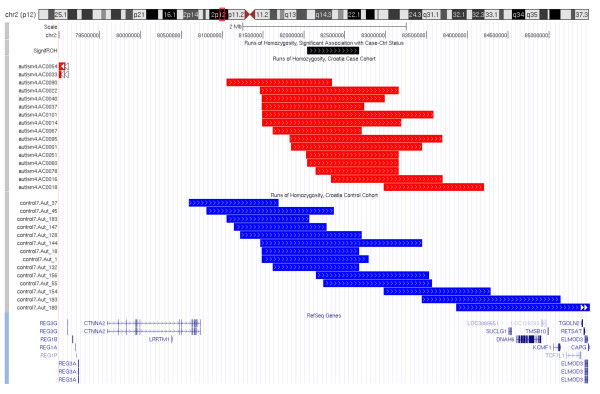
**UCSC Genome Browser genomic annotation around the region chr2:82,040,863-82,677,336**. Genomic annotations of a region where ROH status is associated with case/control status in the Croatia cohort.

**Figure 6:**
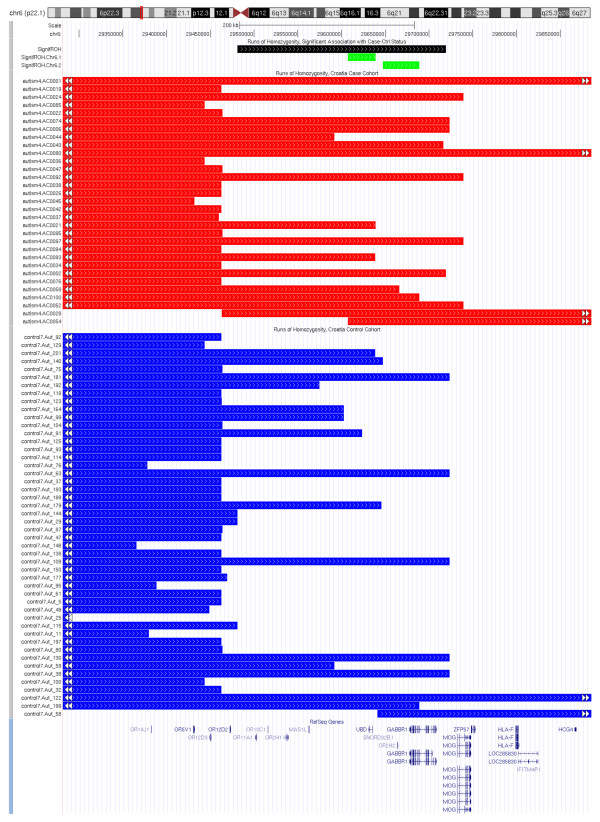
**UCSC Genome Browser genomic annotation around the region chr6:29,481,192-29,719,410**. Genomic annotations of a region where ROH status is associated with case/control status in the Croatia cohort.

ROH regions were common or specific for a demographic group (Croatia vs. North America) or a disease status (ASD vs. controls). Although the longest ROH regions do not need to necessarily coincide with the disease susceptibility, we cannot exclude a possibility that recessive variants, such as single nucleotide or copy number variants within these regions contribute to disease susceptibility. To identify which regions contribute to the significant difference between Croatia case and control groups, we sorted the 7,740 endpoints of all autosomal ROHs across Croatia case and control groups, computed the significance of association between ROH status and case/control status by Fisher's test for each pair of adjacent ROH endpoints, and examined the significance of each such ROH interval where at least 5% (15) individuals have the ROH (Table 5). The most significant ROHs are in two chromosomal regions: chr2:82,040,863-82,677,336 (2p12) and chr6:29,481,192-29,719,410 (6p21.1) (Figure 5 and Figure 6). It is striking these two homozygosity regions are near several genes previously associated with neurological and psychiatric disorders. The chromosome 2 interval (636 Kb) is located downstream of *CTNNA2 *(alpha catenin) and *LRRTM1 *(leucine-rich repeat transmembrane neuronal protein 1), and contains a segment (between SNPs rs2685729 and rs2862972) significantly enriched in ASD subjects (top p = 2.5 × 10^-3^). The *LRRTM1 *gene is associated with left-handedness (patients with autism and many other psychiatric disorders have a higher tendency to be left-handed [39]) and may increase risk of schizophrenia [40,41]. The chromosome 6 interval (238 Kb) is a gene-rich region located distal to the major histocompatibility complex. This ROH is shared by 17 ASD subjects (16.5%) and 19 controls (9.6%) spans 8 genes, including a cluster of olfactory receptors (*OR11A1*, *OR10C1*, *OR2H1*, *OR2H2*), the *MOG *locus, and the *GABBR1 *locus. The *MOG *gene (myelin oligodendrocyte glycoprotein) is a transmembrane protein expressed on the surface of myelin sheath, and may be involved in multiple sclerosis (OMIM#126200) [42,43]; recent studies also found that *MOG *is differentially expressed in prefrontal cortex and temporal lobes in schizophrenic patients [43,44]. The most significant interval (between SNPs rs1233399 and rs29225; top p = 3.4 × 10^-3^) spans the 3' end of the *GABBR1 *(Gamma-aminobutyric acid (GABA) B receptor, 1) and the overlapping *UBD *(Ubiquitin D) loci. *GABBR1 *is highly expressed in nervous systems and has been suggested as a candidate region for neuropsychiatric disorders [45,46]. A recent study showed that the expression levels of GABBR1 are significantly lower in several brain regions in patients with autism compared with healthy controls [47]. Finally, we examined the median IBS distances between the subjects with regions of homozygosity on chromosomes 2 (13 Croatian cases and 10 Croatian controls) and chromosome 6 (17 Croatian cases and 19 Croatian controls) with permutation tests, but found that these subjects were not more closely related than the population average. Thus, the increased homozygosity in Croatia ASD cases cannot be explained by inbreeding among close relatives in a small number of selected families. However, the observed homozygosity may be the result of shared ancestral alleles from generations back in time.

**Table 5:** Top ROH regions in ROH association analysis

CHR	START	END	SIZE (KB)	#SNPs	#ROH&Case	#Case	#ROH&Ctrl	#Ctrl	#ROH	#Samples	OR	P
2	82040863	82067472	26610	3	11	103	8	198	19	301	2.83	0.0424
2	82067472	82143271	75800	11	11	103	7	198	18	301	3.25	0.0195
2	82143271	82146419	3149	3	12	103	7	198	19	301	3.58	0.0105
2	82146419	82235100	88682	18	12	103	8	198	20	301	3.12	0.0153
2	82235100	82276290	41191	6	12	103	9	198	21	301	2.76	0.0305
2	82276290	82330384	54095	4	12	103	8	198	20	301	3.12	0.0153
2	82330384	82342863	12480	2	13	103	8	198	21	301	3.42	**0.0081**
2	82342863	82367354	24492	7	12	103	8	198	20	301	3.12	0.0153
2	82367354	82677336	309983	20	12	103	7	198	19	301	3.58	0.0105
2	82677336	82706135	28800	3	12	103	5	198	17	301	5.06	**0.0025**
2	135475565	135478814	3250	2	4	103	21	198	25	301	0.34	0.0488

4	34049422	34100634	51213	8	30	103	37	198	67	301	1.78	0.0421
4	34100634	34156199	55566	7	30	103	36	198	66	301	1.85	0.0393
4	34156199	34179397	23199	5	29	103	35	198	64	301	1.82	0.0386
4	34378375	34397251	18877	3	26	103	30	198	56	301	1.89	0.0420
4	34531827	34538866	7040	2	17	103	16	198	33	301	2.24	0.0326

5	42508871	42588188	79318	9	11	103	8	198	19	301	2.83	0.0424

6	29481192	29574935	93744	17	16	103	15	198	31	301	2.24	0.0442
6	29574935	29591890	16956	7	16	103	14	198	30	301	2.41	0.0255
6	29591890	29602876	10987	3	15	103	13	198	28	301	2.42	0.0348
6	29602876	29607484	4609	2	15	103	11	198	26	301	2.89	0.0156
6	29607484	29623781	16298	3	16	103	11	198	27	301	3.11	**0.0056**
6	29623781	29638829	15049	5	16	103	10	198	26	301	3.44	**0.0040**
6	29638829	29639232	404	3	15	103	9	198	24	301	3.56	**0.0034**
6	29639232	29641274	2043	2	14	103	9	198	23	301	3.29	0.0101
6	29641274	29645954	4681	4	14	103	10	198	24	301	2.95	0.0131
6	29645954	29647461	1508	2	14	103	9	198	23	301	3.29	0.0101
6	29647461	29666169	18709	6	14	103	8	198	22	301	3.72	**0.0042**
6	29666169	29689020	22852	11	13	103	8	198	21	301	3.42	**0.0081**
6	29689020	29716673	27654	2	12	103	7	198	19	301	3.58	0.0105
6	29716673	29719410	2738	2	11	103	7	198	18	301	3.25	0.0195
6	29719410	29723801	4392	3	10	103	7	198	17	301	2.92	0.0356
6	61949597	62025463	75867	2	11	103	7	198	18	301	3.25	0.0195
6	62030461	62041958	11498	4	12	103	9	198	21	301	2.76	0.0305

7	69474084	69475255	1172	2	2	103	16	198	18	301	0.23	0.0392

8	51323785	51339189	15405	2	7	103	30	198	37	301	0.41	0.0416

12	33162179	33170263	8085	2	10	103	6	198	16	301	3.43	0.0266
12	33170263	33329348	159086	27	10	103	7	198	17	301	2.92	0.0356
12	33329348	33331881	2534	2	11	103	7	198	18	301	3.25	0.0195
12	33331881	33335472	3592	2	12	103	8	198	20	301	3.12	0.0153
12	33335472	33349548	14077	2	13	103	11	198	24	301	2.45	0.0426
12	49797511	49861882	64372	6	10	103	7	198	17	301	2.92	0.0356

Several studies have shown a role for copy number variation in predisposing individuals to complex diseases [48], including ASD [15,49-52]. To address this, we next analyzed CNV calls from the high-density SNP genotyping data in Croatia cases and control subjects. We used PennCNV [53], a high-resolution CNV detection method, to call CNVs from the signal intensity data. We identified 2,303 CNVs (using a 10-SNP threshold) in 304 subjects passing the quality control criteria (Table 6). The median size of CNVs is 71.4 Kb, while the mean size of CNVs is 131.4 Kb. Due to the relatively small sample size, we do not expect that we could detect association of specific CNVs with disease status; instead, we focused on large and rare CNVs observed in cases but not controls, since they are more likely to be disease-related. For example, a recent study identified large (>500 Kb) CNVs to be especially pathogenic [54]; in addition, a CNV study on schizophrenia demonstrates that CNVs >500 Kb tend to be more prevalent in cases versus controls [55-57], and that CNVs >2 Mb are observed exclusively in cases [15,16].

**Table 6:** Summary of the CNV analysis.

CNV size	Croatian ASD (n = 103)						Croatian controls (n = 197)									
	
	all		Cn = 1		Cn = 3		all		cn = 0		cn = 1		cn = 3		cn = 4	
>500 Kb	14	(13.59%)	7	(6.80%)	7	(6.80%)	18	(9.14%)	1	(0.51%)	4	(2.03%)	12	(6.09%)	1	(0.51%)
>1 Mb	8	(7.77%)	4	(3.88%)	4	(3.88%)	8	(4.06%)	0	(0.00%)	3	(1.52%)	4	(2.03%)	1	(0.51%)
>2 Mb	5	(4.85%)	2	(1.94%)	3	(2.91%)	1	(0.51%)	0	(0.00%)	0	(0.00%)	1	(0.51%)	0	(0.00%)

Examining 299 unrelated individuals passing QC, we identified a total of 32 CNVs >500 Kb, including 20 duplications and 12 deletions (Table 7 and Table 8). These large chromosomal rearrangements, detected in DNA samples isolated from blood (rather then cell lines), were confirmed through signal intensity data for the entire chromosome in the Illumina BeadStudio software (data not shown). Among them, 5/103 cases (4.85%) and 1/197 controls subject (0.49%) have CNVs greater than 2 Mb. An exceptionally large CNV was detected in an ASD female that harbored an 18 Mb duplication on chromosome 7 (7q21.11-21.3), which includes several known neurodevelopmental genes (*PCLO*, *SEMA3A*, *SEMA3C*, *SEMA3D *and *SEMA3E*). A duplication of this chromosomal region has been associated with intellectual disability and multiple congenital anomalies [58]. Another female has a 10 Mb deletion on chromosome 11 (11q24.2-q25). The Jacobsen syndrome (MIM147791) is a contiguous gene syndrome caused by terminal deletions of the long arm of chromosome 11 and typically associated with growth and psychomotor retardation and characteristic facial dimorphism [59].

**Table 7:** Large CNVs in the Croatia dataset: CNVs longer than 500 Kb in Croatia Cases.

Chromosomal Region (Cytogenetic Coordinate)	**Copy No**.	Length (Kb)	No. SNPs	Start SNP	End SNP	Genes	Individual	Sex	Age
chr1:144943150-145824905 (1q21.1)	3	882	207	rs6656361	rs11240147	ACP6, BCL9, CHD1L, FMO5, GJA5, PRKAB2	AC0037	m	31
chr2:129716352-130250751 (2q21.1)	1	534	114	rs1251175	rs7578253	HS6ST1 (923711 bp upstream); RAB6C (202954 bp downstream)	AC0068	m	9
chr2:199943597-201740672 (2q33.1)	1	1,797	324	rs13028839	rs10200857	AOX1, BZW1, C2orf47, C2orf60, CFLAR, CLK1, FAM126B, FLJ38973, KCTD18, LOC26010, NDUFB3, NIF3L1, ORC2L, PPIL3, SATB2, SGOL2	AC0086	m	11
chr4:189924391-191164126 (4q35.2)	1	1,240	174	rs4863387	rs13120250	FRG1, TUBB4Q	AC0025	m	22
chr7:124752465-125591197 (7q31.33)	1	839	132	rs7810309	rs510319	POT1 (395192 bp upstream); GRM8 (274696 bp downstream)	AC0058	f	35
chr7:78246495-97499079 (7q21.11-21.3)	3	18,681	3381	rs13308578	rs6960808	ABCB1, ABCB4, ADAM22, C7orf23, CACNA2D1, CD36, CROT, DBF4, DMTF1, GNAI1, GNAT3, GRM3, HGF, KIAA1324L, MAGI2, MGC26647, PCLO, RUNDC3B, SEMA3A, SEMA3C, SEMA3D, SEMA3E, SLC25A40, SRI, STEAP4, ZNF804BCLDN12, FLJ21062, GTPBP10, PFTK1, STEAP1, STEAP2, ZNF804BACN9, AKAP9, ANKIB1, ASB4, ASNS, BET1	AC0015	m	20
chr10:41756307-42461241 (10q11.1-11.21)	3	705	49	rs10909011	rs158389	ZNF33B	AC0061	m	17
chr10:54450683-58765948 (10q21.1)	1	4,315	951	rs2249349	rs2928464	PCDH15, ZWINT	AC0045	m	33
chr11:124367724-134445626 (11q24.2-q25)	1	10,078	2842	rs6590113	rs11224228	ACAD8, ACRV1, ADAMTS15, ADAMTS8, APLP2, B3GAT1, BARX2, C11orf38, C11orf45, CCDC15, CDON, CHEK1, DCPS, DDX25, EI24, ETS1, FAM118B, FEZ1, FLI1, FOXRED1, GLB1L3, HNT, HYLS1, IGSF9B, JAM3, KCNJ1, KCNJ5, KIRREL3, LOC219854, LOC89944, NCAPD3, NFRKB, OPCML, P53AIP1, PATE, PKNOX2, PRDM10, PUS3, RICS	AC0052	f	26
chr12:37835456-42584684 (12q12)	3	4,538	976	rs11170890	rs4488262	ABCD2, C12orf40, KIF21A, LRRK2, SLC2A13CNTN1ADAMTS20, GLT8D3, IRAK4, PDZRN4, PPHLN1, PRICKLE1, PUS7L, TMEM117, TWF1, YAF2, ZCRB1	AC0093	m	17
chr16:15032942-16197033 (16p13.11)	3	1,164	201	rs4985124	rs8056397	ABCC1, ABCC6, C16orf45, C16orf63, KIAA0430, MPV17L, MYH11, NDE1, NTAN1, PDXDC1, RRN3	AC0003	m	31
chr16:21482719-29234430 (16p12.2-11.2)	3	7,175	1266	rs13339281	rs7500911	C16orf65, CDR2, CHP2, COG7, DCTN5, EARS2, EEF2K, ERN2, GGA2, HS3ST2, IGSF6, LOC23117, METTL9, NDUFAB1, OTOA, PALB2, PLK1, POLR3E, PRKCB1, SCNN1B, SCNN1G, UBFD1, UQCRC2, USP31, VWA3ACACNG3, PRKCB1, RBBP6AQP8, ARHGAP17, LCMT1, SLC5A11, ZKSCAN2HS3ST4APOB48R, ATP2A1, ATXN2L, C16orf82, CCDC101	AC0088	m	15
chr17:28980655-29960126 (17q12)	3	979	350	rs11657037	rs11657603	ACCN1, CCL1, CCL11, CCL13, CCL2, CCL7, CCL8, FLJ44815, TMEM132E	AC0100	m	13
chr22:48833840-49524956 (22q13.33)	1	691	126	rs137916	rs2285395	ACR, ADM2, ARSA, CHKB, CPT1B, ECGF1, FAM116B, HDAC10, KLHDC7B, LOC164714, LOC440836, MAPK11, MAPK12, MAPK8IP2, MIOX, MLC1, MOV10L1, NCAPH2, PANX2, PLXNB2, SAPS2, SBF1, SCO2, SELO, SHANK3, TMEM112B, TRABD, TUBGCP6	AC0058	f	35

**Table 8:** Large CNVs in the Croatia dataset: CNVs longer than 500 Kb in Croatia Controls.

Chromosomal Region (Cytogenetic Coordinate)	**Copy No**.	Length (Kb)	No. SNPs	Start SNP	End SNP	Genes	Individual	Sex	Age
chr2:836164-1827317 (2p25.3)	3	991	202	rs4533500	rs11127313	LOC391343, MYT1L, PXDN, SNTG2, TPO	Aut_26	m	28
chr3:186745-1313259 (3p26.3)	3	1,127	339	rs6442427	rs1353825	CHL1, CNTN6	Aut_46	f	36
chr3:57010-1122201 (3p26.3)	3	1,065	267	rs1516321	rs6797539	CHL1, CNTN6	Aut_99	m	14
chr4:188763338-190322725 (4q35.2)	1	1,559	333	rs13147499	rs13106777	FLJ25801, TRIML1, ZFP42	Aut_37	m	27
chr4:190385789-190982886 (4q35.2)	3	597	69	rs10446846	rs6844114	TRIML1 (1080146 upstream); FRG1 (116082 downstream)	Aut_141	m	20
chr5:104486852-105008254 (5q21.2-21.3)	3	521	58	rs294152	rs6872786	NUDT12 (1560463 upstream); EFNA5 (1735996 downstream)	Aut_36	f	29
chr7:49000866-49879997 (7p12.3-12.2)	3	879	165	rs1004168	rs6963083	VWC2	Aut_203	f	20
chr7:79347222-81014740 (7q21.11)	1	1,668	336	rs6949571	rs2909580	CD36, GNAI1, GNAT3, SEMA3C	Aut_65	m	8
chr8:4005541-4886423 (8p23.2)	1	881	781	rs7006672	rs7815159	CSMD1	Aut_38	f	25
chr8:85925158-87860757 (8q21.2-21.3)	1	1,936	257	rs4740033	rs11781187	ATP6V0D2, C8orf59, CA1, CA13, CA2, CA3, CNGB3, CPNE3, E2F5, FAM82B, LRRCC1, PSKH2, RALYL, REXO1L1, SLC7A13, WWP1	Aut_114	f	29
chr9:22934927-23536461 (9p21.3)	3	602	136	rs1463014	rs7028484	DMRTA1 (492455 upstream); ELAVL2 (143644 downstream)	Aut_54	m	27
chr12:33415349-34701470 (12p11.1)	4	1,286	104	rs1905414	rs9706509	ALG10, SYT10	Aut_39	m	23
chr15:70783089-73316235 (15q24.1-24.2)	3	2,533	382	rs11072382	rs1565496	ADPGK, ARID3B, BBS4, C15orf17, C15orf39, CCDC33, CD276, CLK3, COX5A, CPLX3, CSK, CYP11A1, CYP1A1, CYP1A2, EDC3, GOLGA6, HCN4, ISLR, ISLR2, LMAN1L, LOC283677, LOC388135, LOXL1, MPI, NEO1, NPTN, PML, PPCDC, RPP25, SCAMP2, SCAMP5, SEMA7A, STOML1, STRA6, TBC1D21, UBL7, ULK3	Aut_10	m	48
chr16:76525505-77164850 (16q23.1)	3	639	235	rs387138	rs2346008	CLEC3A, KIAA1576, WWOX	Aut_148	m	38
chr22:17690812-18613429 (22q11.21)	3	923	248	rs982520	rs854971	ARVCF, C22orf25, C22orf29, CDC45L, CLDN5, COMT, DGCR8, GNB1L, GP1BB, HIRA, HTF9C, LOC128977, MRPL40, RANBP1, RTN4R, SEPT5, TBX1, TXNRD2, UFD1L, ZDHHC8	Aut_94	m	6
chr22:19102598-19665559 (22q11.21)	3	563	97	rs738089	rs7292968	AIFM3, CRKL, KLHL22, MED15, PI4KA, SCARF2, SERPIND1, SNAP29	Aut_94	m	6
chrX:6468166-8112188 (Xp22.31)	3	1,644	190	rs6654819	rs2278935	HDHD1A, PNPLA4, STS, VCX, VCX2	Aut_157	f	41
chrX:93063380-93732495 (Xq21.32-21.33)	0	669	88	rs6615574	rs5950049	FAM133A (209463 upstream); DIAPH2 (2093870 downstream)	Aut_155	m	38

**Figure 7:**
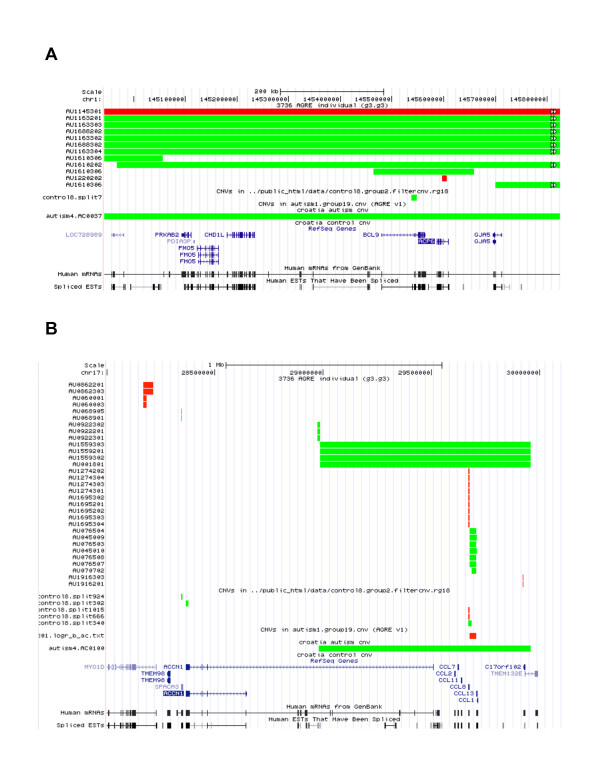
**CNV status of AGRE, NINDS control, and Croatia ASD/control cohorts**. (A) CNVs around four candidate genes (*ACP6*, *CHD1L*, *FMO5*, and *PRKAB2) *in 882 kb duplication on 1q21.1 (chr1:144,943,150-145,824,905). (B) Duplication of size 979 kb around the neuronal amiloride-sensitive cation channel gene (*ACCN1) *on Chr 17q11.2-q12 (chr17:28,000,000-30,100,000).

Three of the ASD cases have multiple large rearrangements; one adult male harbors two chromosomal rearrangements (separated by 117 Mb) on chromosome 1, a 335 Kb deletion on 1p35.3 and a 882 Kb duplication on 1q21.1 (Figure 7a). In a second individual (adult female) we identified two distinct large CNVs: a 838 Kb deletion in an intergenic region between *GRM8 *and *POT1 *on 7q31.33 (chr7:124,752,465-125,591,197) and a 691 Kb deletion at 22q13.33 (chr22:48,833,840-49,524,956). This deletion encompasses the *SHANK3 *gene and cytogenetic rearrangements affecting this gene have been previously associated with ASD [60,61]. It has been previously reported that the 22q13 deletion syndrome, also known as Phelan-McDermid syndrome (MIM606232), is characterized by severe neonatal hypotonia and global developmental delay, normal to accelerated growth, absent to severely delayed speech, and minor dysmorphic features [62,63]. The third individual (male child) with multiple large rearrangements harbors a 534 Kb intergenic deletion at 2q21.1 (chr2: 129,716,352-130,250,751) and a 400 Kb duplication at 3q29 (chr3:195,987,870-196,387,903). These large structural variants, especially those found on multiple chromosomes illustrate the need to combine high-density genotype analysis with karyotype analysis to test for the presence of balanced translocations and inversions.

In contrast to large rearrangements encompassing >10 Mbs that were observed in ASD cases, the largest rearrangement observed in control subjects is a 2.5 Mb duplication at 15q24.1-q24.2 (chr15:70,783,089-73,316,235), a known microdeletion/duplication region [64]. Also, we found one control subject to have two duplications within the DiGeorge (MIM188400) and Velocardiofacial (MIM192430) syndrome region on chromosome 22 (22q11.21), a known pathogenic rearrangement hotspot where large (>1.5 Mb) deletions are pathogenic [65]. These duplications (922 Kb in chr22:17,690,812-18,613,429 and 563 Kb in chr22:19,102,598-19,665,559) are separated by 489 Kb. The observation that we see more large CNVs associated with ASD subjects (>2 Mb: 5 cases, 1 control; Fisher's test; p = 0.019) suggests these large rearrangements may be rare ASD susceptible variants; follow-up investigations on larger cohorts will be necessary.

## Conclusions

In this paper, we report analysis of a genome-wide high-density genotype study for ASD in Croatia. The dataset, although small, provides a distinctive opportunity to evaluate structural variation associated with a complex disease such as ASD in a genetically distinct population. In addition to the analysis of ASD, high-density genotype data from a well-defined geographic area of Croatia permitted also the study of the subjects' genetic substructure and diversity. We asked whether the subjects included in this study which were collected throughout Croatia, show a lower level of genomic diversity in comparison to subjects from other defined demographic areas. Addressing this issue may suggest that a set of 100 Croatian ASD subjects harbor a smaller set of susceptibility loci in comparison a randomly selected set of ASD subjects from a larger area. The Croatian samples represent a genetic continuum of relatedness with a subset of individuals closer to those from several regions in Europe, probably due to numerous historic migrations. The impact of fine-scale population history on the genetic substructure of individuals from Croatia has recently been evaluated using a small set of selected loci [21]. To our knowledge, this study is the first to provide a genome-wide view of the genetic composition of the Croatia population.

Although our study represents the first high-resolution genomic analysis of subjects with ASD in Croatia, several caveats need to be addressed. Apart from an obviously limited sample size, the classification of ASD subjects in this pilot project has been established using the DSM-IV criteria. Due to phenotypic heterogeneity of ASD and our finding of a wide-range of novel, and in some cases, exceptionally large CNVs, there is a need for accurate clinical diagnostic criteria for the diagnosis of ASD and related neurodevelopmental syndromes. Currently no other diagnostic tools were available in Croatian; in order to compare our findings to those in other large-scale genetic studies it will be necessary to translate gold standard diagnostic tools such as ADOS [66] and ADI-R [67] to Croatian and many other languages.

We used PennCNV to call copy number variations, though other CNV calling programs such as QuantiSNP [68] and cnvPartition (a built-in software tool in the Illumina BeadStudio software)are available An advantage of using multiple CNV calling programs is to reduce false positives, namely one can focus on CNVs returned by multiple programs and expect them to be "more reliable". Since our study focused on large CNVs (>500 Kb) we were able to validate these variants by visual inspection of the raw intensities(data not shown).

We found that runs of homozygosity are more frequent in Croatia ASD subjects than either Croatia or NINDS control cohorts. Given the low frequency of subjects with ROH, larger follow-up studies are necessary to confirm the findings. Our findings suggest there is a high chance that homozygosity mapping based on family studies can discover rare recessive variants. Our finding that several ASD subjects harbor exceptionally large chromosomal rearrangements previously associated with distinct clinical syndromes such as Velocardiofacial, Jacobsen, Phelan-McDermid, or 3q29 microdeletion/duplication syndromes (MIM609425; MIM611936) lends support for cytogenetic or high-density genotype testing for ASD subjects with complex clinical manifestation [69]. Furthermore, recent findings that seemingly diverse neurodevelopmental and neurological disorders are often associated with the same chromosomal rearrangement(s) provide support for in-depth phenotype analysis and longitudinal clinical evaluation of entire families. These family-based studies may also reveal whether observed structural variants are *de novo *events or rather inherited and present in additional family members. We suggest that deeper population screens combined with family-based genetic analyses will lead to improved understanding of joint actions of associated loci and molecular basis of neurodevelopmental disorders in populations worldwide.

## Methods

### Subjects and DNA Preparation

The patient group consisted of 103 individuals (81 male, 22 female, mean age 21.5 ± 10.3, ranging from 4 to 45) recruited from the Centers for Autism in Zagreb, Rijeka and Split (Republic of Croatia) and diagnosed with autism spectrum disorders according to DSM-IV criteria. The control group consisted of 203 healthy blood donors (146 male, 57 female, mean age 32.5 ± 8.06, ranging from 19 to 45) [30] with no history of mental illnesses, behavioral disorders, or substance abuse. All subjects were of Croatian (southern Slavic) origin. After an informative talk, a written consent for inclusion in the study was obtained from the control subjects and from the patients' parents. The study has been carried out in accord with the Declaration of Helsinki, and was approved by the Ethics Committee of the Medical Faculty of the University of Zagreb.

Blood sampling was performed in the Centers for Autism (ASD cases) and in the Croatian Institute of Transfusion Medicine (control subjects) between 9 and 11 a.m. Either 2 or 5 ml of venous blood, depending on the age of the participants, was collected into vacutainers containing EDTA anticoagulant. DNA was isolated from the whole blood using a DNA isolation kit for mammalian blood (Boehringer Manheim, Germany).

### Datasets and Preprocessing

The genomic DNA from blood was used to obtain genotypes by the Illumina HumanHap550 version 3 high-density array with 561,446 SNP markers. Coordinates of SNPs as well as genomic annotations are based on NCBI human genome build 36.3, (UCSC Genome Browser Release 18). The genotyping experiments were performed at the Center for Applied Genomics, Children's Hospital of Philadelphia, as previously described [70]. The raw genotyping signal data were processed by the Illumina BeadStudio software and converted to normalized signal intensity values, represented as Log R Ratio (LRR) and B Allele Frequency (BAF). Due to the presence of "genomic wave patterns" in some of the genotyped samples, we applied a data pre-processing protocol [71] to increase the signal-to-noise ratio of the LRR values for all samples.

We analyzed three genome-wide datasets: Croatia Autism cohort (306 subjects), NINDS Control cohort (542 subjects), and genotyping data from the Human Genetic Diversity Panel (1043 subjects, genotyped at Stanford University, downloaded from http://hagsc.org/hgdp/files.html). The quality control (QC) procedure (described in detail in Wang et al. [14] and Bucan et al. [16]), done separately for the three datasets, is as follows: (1) we first discarded SNPs with 5% or more missing genotyping calls; (2) we then recomputed summary statistics for individuals and markers; (3) we retained individuals/markers passing the following threshold: individual - missing genotyping call percentage ≤5%; marker - missing genotyping call percentage ≤5%, minor allele frequency ≥5%. We then merged the Croatia cohort with the NINDS control and HGDP datasets separately into two combined datasets (Croatia samples and HGDP, Croatia samples and NINDS), including all individuals passing QC in their separate datasets, and retaining only markers passing QC in both datasets. Subpopulation membership for HGDP samples were obtained from Supplemental material from Kidd et al. [72]

To detect hidden related samples, we computed $\overset{\wedge }{\pi }$, the estimated probability of IBD between every pair of subjects from the Croatia control and case and NINDS control samples using PLINK. We found 5 pairs of Croatia control samples (but none among the case samples) and 4 pairs of NINDS control samples have $\overset{\wedge }{\pi }$ > 0.25 (siblings or closer). To be more specific, three Croatia control pairs and one NINDS control pair have $\overset{\wedge }{\pi }$ > 0.999 (duplicate genotyping or monozygotic twins), and two Croatia Ctrl pairs and three NINDS control pairs have $\overset{\wedge }{\pi }$ around 0.5 (first-degree relatives). To reduce relatedness, in subsequent analysis, for each pair we removed the one of the two samples with a larger index number in the ROH analysis. Table 1 summarizes the three genome-wide datasets analyzed in this paper.

### IBS distance analysis

We computed the IBS (identity by state) distance between any two subjects using PLINK, and constructed three pairwise distance matrices: Croatia controls and HGDP, Croatia controls and NINDS, Croatia cases and controls. The IBS distance between two subjects is defined as A+B/2, where A and B are the number of SNPs that differ by one and two alleles between the two subjects, respectively. We performed multidimensional scaling using R [73] and plotted the first two coordinates, and built trees using FastME, a distance-based phylogeny reconstruction software [74].

### F_ST _Analysis

To quantitatively describe the genetic difference between two population we next used the F_ST _coefficient (fixation coefficient). This measure combines information across many loci in many individuals and a higher F_ST _value for a locus indicates difference between the genetic compositions in two populations, whereas F_ST _= 0 implies no discernable difference between two populations at the locus.

We analyzed the F_ST _values using the Croatia control and NINDS control cohorts as follows. We first computed the heterozygosity of each marker (defined as the proportion of heterozygous individuals in the population) using PLINK, then computed the fixation index *F*_*ST *_from the heterozygosity by R by the following steps. (1) Compute the minor allele frequencies p_1_, p_2 _and p for the two subpopulations (with N_1 _and N_2 _subjects) and the whole population (with N = N_1 _+ N_2 _subjects). (2) Compute the expected proportion of heterozygotes H_T _= p (1-p) assuming the two subpopulations are genetically identical for the marker. (2) Compute the expected proportion of heterozygotes H_S _= (N_1 _p_1 _(1-p_1_) + N_2 _p_2 _(1-p_2_))/N. (3) Compute FST=1−HSHT.

### Runs of homozygosity

Runs of homozygosity (ROHs) are extended genomic regions where the DNA sequences on the two chromosomes are identical, and may be due to identity by descent or gene conversion; since polymorphic sites are all homozygous, SNP arrays can be used to identify such regions. Study of ROHs can provide information on the population structure such as extent of inbreeding, and can be used to find candidate genes in recessive disorders. We computed the distribution of runs of homozygosity for three groups (Croatia case, control, NINDS) using PLINK with the following settings: an interval passes as an ROH if it has 100 or more homozygous SNPs (except at most one SNP), with density at least one SNP every 50 Kb and at most 1 Kb gap between any two neighboring SNPs. We ran Wilcoxon test between every pair of the three groups, as well as Kruskal-Wallis test for comparing three groups simultaneously.

To identify genomic regions where the ROH status correlates with the case/control status, we ran Fisher's test as follows: we first sorted the 7,766 endpoints of all autosomal ROHs across Croatia case and control groups; for each pair of adjacent endpoints, we computed the significance of association between ROH status and case/control status by Fisher's test.

### Correlation between ROH and age

Motivated by the study demonstrating the positive correlation between runs of homozygosity and age in a North American cohort of European descent due to urbanization [35], we examined the correlation between age and ROH frequency and size. Only the 103 ASD subjects have age information. We used age and sex as covariates in a full linear regression model (in R/S-Plus specification):

Y~AGE*SEX

Here the dependent variable can be either the total ROH length, average ROH length, or number of ROH regions in a subject.

### CNVs analysis

A previously described high-resolution CNV detection algorithm, the PennCNV algorithm [53], was used to infer CNVs from the signal intensity data following protocols described in Wang et al. [53] The PennCNV algorithm uses a Hidden Markov model to identify any genomic regions with duplications or deletions by combining signal intensity and SNP allelic ratio distributions. The Illumina BeadStudio software provided visualization tools that were used to display signal intensity data for the entire chromosome.

## Competing interests

The authors declare that they have no competing interests.

## Authors' contributions

MB, DH and HH designed the study. DH, ZBP, and BJ contributed samples. LW, KW, IL, LY, NG, HH and MB participated in the analysis. LW and MB wrote the paper. All co-authors contributed to the preparation of the paper.

## Pre-publication history

The pre-publication history for this paper can be accessed here:

http://www.biomedcentral.com/1471-2350/11/134/prepub
